# When is the Best Time for Corrective Surgery in Patients with Tetralogy of Fallot between 0 and 12 Months of Age?

**DOI:** 10.21470/1678-9741-2018-0019

**Published:** 2018

**Authors:** Izabela F. Martins, Iara C. Doles, Nathalie J. M. Bravo-Valenzuela, Adriana O. R. dos Santos, Marcela S. P. Varella

**Affiliations:** 1 Universidade de Taubaté (UNITAU), Taubaté, SP, Brazil.; 2 Escola Paulista de Medicina, Universidade Federal de São Paulo (EPM-UNIFESP), São Paulo, SP, Brazil.

**Keywords:** Tetralogy of Fallot/Surgery, Infant, Newborn, Infant

## Abstract

**Objective:**

To identify the best time for corrective surgery of tetralogy of Fallot (TF) in children aged 0-12 months and to report the most frequent complications during the first 3 years postoperatively.

**Methods:**

Systematic review of studies published between 2000 and 2017 on corrective surgery for TF. Articles were selected through search of electronic databases (PubMed, SciELO, Scopus, Lilacs, Google Scholar, and Cochrane). Length of stay in intensive care unit, duration of mechanical ventilation, and peri/postoperative complications were analyzed for data discussion and research interpretation.

**Conclusion:**

Definitive corrective surgery is the best alternative, and the earlier it is performed, the lower the occurrence of harmful effects and the greater the chances of cardiorespiratory recovery. This systematic review suggests that the best time to perform definitive corrective surgery for TF in the first year of life is during 3-6 months of age in children with no or mild symptoms. Children with severe symptoms should undergo surgery immediately.

**Table:** 

Abbreviations, acronyms & symbols	
CHD	= Congenital heart disease
ECC	= Extracorporeal circulation
ICU	= Intensive care unit
NYHA	= New York Heart Association
RV	= Right ventricle
TF	= Tetralogy of Fallot

## INTRODUCTION

Tetralogy of Fallot (TF) is the most common cyanogenic congenital heart disease (CHD) occurring after the first year of life and it accounts for approximately 10% of all CHD^[1]^. The clinical presentation is variable and the symptoms, such as progressive cyanosis and reduced physical capacity, are directly related to the obstruction of the right ventricle (RV) outflow tract. The natural history of TF is quite variable and is determined by the type and degree of obstruction of the RV outflow tract. Most patients, such as infants, are asymptomatic, but if they are not treated surgically, only 10% will survive beyond the age of 20 years^[2]^. In contrast, we can expect that 90% of the patients who undergo definitive surgery in childhood survive up to the fifth decade of life. Without surgical correction, consequences of progressive hypoxemia and acquired changes, such as hypoplasia of the pulmonary arteries and RV and development of collateral systemic pulmonary circulation, will occur. These late changes are the reasons why corrective surgery is being performed earlier and earlier.

In general, early surgery is indicated by the severity of the symptoms and associated injuries. However, attacks of hypoxia, which occur more frequently between 2 and 3 months of age and are not related to the degree of pulmonary stenosis, may evolve with cerebrovascular accidents that may lead to neuro and psychomotor developmental sequelae and death. Thus, there is a current trend toward a preference for definitive correction in cases of TF of good anatomy during the first year of life, aiming to reduce the harmful effects of hypoxemia and to improve the physiological outcome^[3]^. However, the best time to perform this operation remains controversial^[4,5]^.

The natural history of TF has undergone major modifications over the last few decades. With the evolution of surgical methods to correct cardiopathies during the neonatal period and the development of specialized intensive care units (ICU), it has been possible to achieve better results at an increasingly earlier age^[6,7]^. These developments directly influenced the earlier indication of correction in children with this condition. In 1973, Barratt-Boyes & Neutze^[8]^ and Starr et al.^[9]^ showed that TF could be corrected with extracorporeal circulation (ECC) in the first year of life with low morbidity and mortality. In 2005, van Arsdell et al.^[10]^ concluded that the best time for correction of TF was between 3 and 11 months of age based on mortality and better physiological outcome. However, Castañeda et al.^[11]^ indicated accentuated hypoplasia of the pulmonary arteries and anomalous origin of the anterior descending coronary artery (originating from the right coronary artery) as risk factors that could contraindicate definitive correction of this CHD during the first year of life.

Given this context, there is a current trend to prefer definitive correction of classic TF during the first year of life instead of correction in two phases (palliative shunt operation and subsequent surgical correction of TF)^[12]^. However, the ideal time for surgical correction of this CHD during the first year of life remains controversial. Age, weight, and clinical condition of the patient, presence or absence of associated extracardiac defects, and experience of the service where the operation will be performed in addition to a careful evaluation of the cardiac anatomy of each patient (characteristics of the RV outflow tract, pattern of coronary circulation, and analysis of the ventricles) are factors that must be considered for an indication of the best time for the correction of this CHD^[13,14]^.

In this study, we systematically reviewed relevant studies published between 2000 and 2017, including those in which definitive correction of TF was performed during the first year of life. We considered peri- and postoperative mortality and complications, such as duration of ECC, arrhythmias, ventricular dysfunction, and pulmonary insufficiency and/or stenosis requiring surgical reintervention, to conclude which is the best time for this operation. Moreover, we discussed the unfavorable factors of the cardiac anatomy and small body surface area (<0.46 m^2^), which are considered risk factors for the correction of classic TF.

## METHODS

### Sources of Electronic Searches

**Fig. 1:**
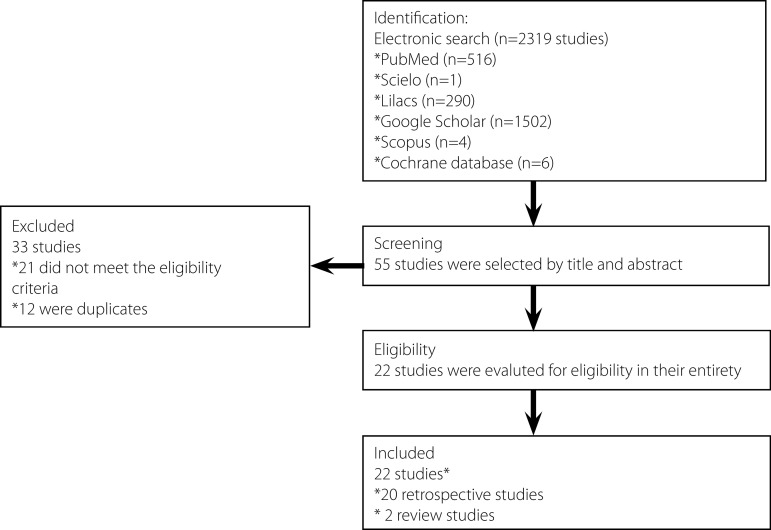
Flowchart of records identified after an electronic search (by title and abstract) of articles published during 2000–2017 on the surgical correction of tetralogy of Fallot performed during the first year of life.

This systematic review was performed on 5 steps according to Khan et al.^[15]^: step 1 – the review question, step 2 – study selection, step 3 – quality assessment of the studies, step 4 – data synthesis, and step 5 – interpreting the findings. The search was based on the following question: “When is the best time for corrective surgery in patients with Tetralogy of Fallot between 0 and 12 months of age”. The electronic search for articles published from 2000 to November 2017 in the PubMed/Medline, SciELO, Lilacs, Google Scholar, Cochrane, and Scopus databases was conducted without language restriction (Figure 1). However, the descriptors were used both in English and in Portuguese: “Tetralogy of Fallot”, “surgery”, “newborn”, and “infant”.

### Study Selection

The titles and abstracts of all publications obtained from the electronic search and their lists of references were screened extensively by the authors. The pre-established inclusion and exclusion criteria were applied, and articles with complete texts were selected to be examined and included in this review. Indeed, the studies selected were assigned according to the research design's quality, such as homogeneous or heterogeneous populations, bias adjustment factors, short or long-term follow-up, and prospective or retrospective study.

### Selection Criteria

Studies published between 2000 and 2017 on corrective surgery in patients younger than 12 months of age who presented with the classic TF were included. Patients with pulmonary atresia or other cardiac or associated extracardiac malformations were excluded. Duplicate studies and those with possible design bias or poor-quality design on safety were excluded (Figure 1). Indeed, cases studies and opinion of experts were not included.

## RESULTS

### Selected Studies

The electronic search retrieved a total of 2,316 records that were displayed based on their titles and abstracts. Of these, 55 were selected based on their titles and abstracts. Thirty-three publications were excluded because they did not meet the selection criteria, remaining 22 articles eligible for the study (Figure 1).

### Studies' Characteristics

There was a total of 20 cohort studies and two bibliographical reviews (Table 1). Twenty articles were published in English and two in Portuguese. Studies were conducted in the United States of America, four in Germany, four in Canada, in the United Kingdom, two in Brazil, one in Turkey, one in Austria, and one in Korea.

**Table 1:** Main studies indicating the optimal age (from birth until 12 months) for correction of tetralogy of Fallot (TF).

Author, year	Study design	Study period	Number of surgical complete repairs	Early surgical mortality	Conclusion (optimal age for surgical repair of TF)
Barron ^[5]^, 2013	Retrospective Multicenter	2002 to 2007	3000 178 neonatal repairs	1.9 to 3% 7.8% (neonates)	Optimal age: 3 to 9 months
Kirsch et al.^[30]^, 2014	Retrospective Single-center	1995 to 2009	277	-	Optimal age: 3 to 6 months
Gerrah et al.^[14]^, 2015	Retrospective Single-center	2005 to 2013	51	-	Primary surgical repair for correction of TF has a more favorable outcome than palliation in children weighing less than 4 kg
Park et al.^[17]^, 2010	Retrospective Single-center	2000 to 2008	13	-	Optimal age: less than 3 months, earlier surgical correction of TF may be better for the pulmonary artery growth
Kantorova et al.^[31]^, 2008	Retrospective 2 institutions	1996 to 2006	61	1.63%	Optimal age for asymptomatic patients: more than 3 months. Optimal age for symptomatic patients: as early as possible
Alexiou et al.^[22]^, 2001	Retrospective Single-center	1974 to 2000	89	1.1%	Optimal age: within 12 months
Egbe et al.^[6]^, 2014	Retrospective Single-center	2001 to 2012	97	-	Age and weight are independent predictors of morbidity
Van Arsdell et al.^[4]^, 2000	Retrospective Single-center	1996 to 1998	227	(0 to 12m)	Optimal age: 3 to 11 months
Van Arsdell et al.^[10]^, 2005	Review Single-center	1996 to 2004	357	0.6%	Optimal age: 3 to 6 months
	
Parry et al.^[25]^, 2000	Retrospective Single-center	1992 to 1999	42	-	Optimal age: before 3 months
Moraes Neto et al.^[28]^, 2008	Retrospective Single-center	1996 to 2004	67	2.98%	Optimal age: before 12 months
Moraes Neto et al.^[27]^, 2000	Retrospective Single-center	1986 to 1999	30	6.6%	Optimal age: before 12 months. Expansion of the outflow tract involved in right ventricular obstruction and transannular patch as a risk factor in body surface areas <0.48 m²
Kaulitz et al.^[18]^, 2001	Retro- and prospective Single-center	Follow-up 80,4 ± 24 months	62	4.8%	Optimal age: from 3 to 12 months
	
Steiner et al.^[13]^, 2014	Retrospective Multicenter	2004 to 2010	4698	1.3%	Optimal age: from 3 to 12 months
				6.4% (neonates)	
Starr^[29]^, 2010	Review	1952 to 2010	2715	50's: 60% Early 60's: 5-14% 70's to 2009: <2%	From 70's to present: complete repair of TF is performed before 6 months of age with low mortality.
Kolcz & Pizarro^[16]^, 2005	Retrospective Single-center	1998 to 2004	66	4.5%	Elective repair of TF in neonates has excellent results. Preoperative weight < 2.5 kg and small left pulmonary artery size are associated with higher incidence of reintervention
Bakhtiary et al.^[21]^, 2013	Retrospective Single-center	1998 to 2009	120	-	Optimal age: up to 4 months
Al Habib et al.^[7]^, 2010	Retrospective Multicenter	2002 to 2007	2534	1.3%	Optimal age: before 12 months
				7.8% (neonates)	
Wilder et al.^[12]^, 2017	Retrospective Single-center	2000 to 2012	453	0.67%	Optimal age: 3 to 9 months
Gerling et al.^[20]^, 2009	Retrospective Single-center	1992 to 2003	124	4.8%	Optimal age: from 3 to 12 months
Ooi et al.^[33]^, 2006	Retrospective Single-center	1997 to 2003	52	1.9%	Optimal age: 3 to 6 months
Tamesberger et al.^[34]^, 2008	Retrospective Single-center	1995 to 2006	90	___	Neonatal primary surgical repair is associated with more frequent use of transannular patch and reinterventions

In the 20 cohort studies, 6,801 definitive correction surgeries for TF were performed, all in patients younger than 12 months of age. The mean mortality rates of the 22 studies (after the 50's) were 1.99% and 4.55% for neonates.

The main perioperative complications found were enlargement of the outflow tract, most commonly the placement of a transannular patch (44.5% in patients from 1 to 12 months and 66 to 100% in neonates), prolonged ECC, and cardiorespiratory arrest during surgery.

The most frequently reported postoperative complications after 3 years of follow-up were arrhythmia, ventricular dysfunction, and pulmonary insufficiency, ranging from mild to severe.

## DISCUSSION

Over the past two decades, the number of definitive repairs performed has increased significantly, with early repair becoming increasingly common. All the 22 articles stated that definitive surgery for TF performed in patients younger than 1 year of age offers numerous advantages over that performed in patients after this age.

Early normalization of cardiovascular physiology with consequent reduction in secondary damage, reduction in the harmful effects of chronic hypoxemia present in patients with TF, and minimization of ventricular hypertrophy are some of the benefits of performing surgery in patients younger than 1 year of age. In addition, the morbimortality of systemic pulmonary shunts has been practically eliminated.

Several authors such as Kolcz and Pizarro^[16]^ advocate early repair because it favors the growth of the pulmonary artery, which becomes capable of providing blood flow and alveolar growth to the child. Two other authors, Park et al.^[17]^ and Kaulitz et al.^[18]^, demonstrated growth and increased diameter of the pulmonary arteries when the first surgery to repair TF was performed during the first year of life. In the study by Park et al.^[17]^, the growth of the pulmonary arteries after restoration of blood flow was evaluated based on the Nakata index using transthoracic echocardiography instead of cardiac catheterization to conduct the measurements (Nakata index = ratio between the sum of the areas of the pulmonary arteries and the body surface area, expressed in mm^2^ per m^2^)^[19]^. This study, using the quantitative method, corroborated the conclusion that early surgical correction of TF in a single stage contributes to a greater growth of pulmonary arteries than palliative surgery and in the second stage of correction, it reduces the risk of surgical reintervention or short-term reoperation^[17]^.

Mortality in children with definitive early repair is markedly lower. A study conducted by Gerling et al.^[20]^ in Germany reported a mortality of 3.2% in patients younger than 12 months of age compared with 12.9% in those older than that.

Another study, from Germany, by Bakhtiary et al.^[21]^ presented the observed long-term benefits. It was conducted with 120 children who underwent surgery at younger than 1 year of age. It was shown that 80% of the children were in New York Heart Association (NYHA) class 1 at the end of the study and remained off antiarrhythmic drugs at 10 years of postoperative follow-up. Similarly, in a study by Alexiou et al.^[22]^, 86 of the 89 patients who underwent surgery before 12 months of age remained in NYHA 1 without the use of antiarrhythmic drugs in the postoperative period.

None of the studies reported an increased mortality in surgical reintervention of patients younger than 6 months of age. However, of the 20 evaluated articles, 50% reported a higher surgical risk in children younger than 3 months of age. Vohra HA et al.^[23]^ and van Dongen et al.^[24]^ showed that patients younger than 3 months of age had longer hospital and ICU stays, a greater need for the use of inotropes and for volume replacement, and a higher incidence of organ dysfunction.

Park et al.^[17]^ and Parry et al.^[25]^ demonstrated that an early repair of TF may allow normal RV outflow tract growth thereafter. However, the need for enlargement of the outflow tract was more often observed in patients younger than 3 months of age when comparing the studies reviewed. In the study conducted at the Slovak Pediatric Heart Center, in Bratislava, and at the University Hospital Hamburg-Eppendorf, 61 children underwent definitive surgery and 85% of those younger than 3 months of age required the placement of a transannular patch as opposed to 61% of those older than that^[26]^. On the other hand, 35% of the articles in the review showed that the age limit for good results, both in short and long terms, is 6 months.

According to a cohort study by Moraes Neto et al.^[27]^, the need for enlargement of the outflow tract may compromise the late postoperative period, and the placement of a transannular patch is a significant risk factor in children with body surface area < 0.48m². Based on the evaluation criteria of perioperative complications in that review, the necessity of enlargement of the outflow tract is a factor to be considered when determining the best age for surgery^[28]^.

In the cohort study by Moraes Neto et al.^[27]^, 56 children were divided into two groups, with one of the groups comprising children between 3 and 11 months of age who underwent definitive corrective surgery. In this group, 73.3% of the patients required enlargement of the right ventricular outflow tract and 50% of them required transannular patch repair. Of these 50%, 10 were children older than 6 months of age and only 5 were younger than 6 months of age.

Overall mortality was higher in children older than 6 months of age. In a cohort study presented by Steiner et al.^[13]^, a mortality rate of 0.7% was observed in children between 3 and 6 months of age as compared with 6.4% in those younger than 30 days of age, 1.9% in those between 31 days and 3 months of age, and 1.1% in those older than 6 months of age.

Van Arsdell et al.^[10]^ demonstrated that surgery performed at 6 months of age is very well tolerated, with little development of restrictive physiology, minimal need for prolonged ventilation time, and no development of ascites. The deaths reported were of patients with primary repair after 12 months of age. They also state that neonatal repair does not appear to be the best strategy because of greater development of physiological restriction, higher incidence of stroke, and, in general, longer mechanical ventilation and ICU length of stay.

In a study titled “Tetralogy of Fallot: Yesterday and Today,” the author, Starr JP, supports a concept that has been around since 1970: the best age for definitive repair is before 6 months of age^[29]^. This study shows a mortality rate of 2% and a long-term survival rate ranging from 58% to 90%. In addition, the author reports that initial survival following definitive repair of TF ranges from 98% to 100%. The study does not recommend exceeding a maximum of 12 months of age. Indeed, Kirsch et al.^[30]^ demonstrated that the best age for surgical correction of TF is between 3 and 6 months and Kantorova et al.^[31]^ concluded that the optimal age for the complete surgical repair for asymptomatic TF patients is after 3 months.

Barron et al.^[5]^ and Steiner et al.^[13]^ demonstrated that the mortality for primary repair was higher in neonates with TF when compared to infants older than 30 days of age^[15]^. Controversially, there were no differences in post-operative age-related mortality in the studies conducted by Loomba et al.^[32]^ and Ooi et al.^[33]^. Meanwhile, Van Arsdell et al.^[10]^ and Tamesberg et al.^[34]^ showed that neonatal surgical primary repair for TF was associated with longer total hospital length of stay, more frequent use of transannular patch, and reinterventions. Furthermore, preoperative weight < 2.5 kg and small left pulmonary artery size are associated with higher incidence of reintervention^[16]^.

## CONCLUSION

With advances in medicine, the guarantee of quality of life for children diagnosed with TF has become a reality. Definitive surgical correction is the best choice, and the earlier the treatment, the lower the occurrence of harmful effects and the greater the chance of cardiorespiratory recovery.

The best age for performing definitive corrective surgery for TF is still a topic of discussion in cardiology and pediatric cardiac surgery. Based on this careful systematic review – our analysis of all the articles, considering a postoperative period of up to 3 years, length of stay in ICU, duration of mechanical ventilation, peri- and postoperative complications, need for reoperation, and mortality –, we concluded that the best age for elective definitive surgical correction of TF is between 3 and 6 months of age in asymptomatic and mildly symptomatic children. Children with severe symptoms should undergo surgery immediately, regardless of age.

**Table:** 

Authors' roles & responsibilities	
IFM	Collecting the and interpretation of data for the work; final approval of the version to be published
ICD	Collecting the and interpretation of data for the work; final approval of the version to be published
NJMBV	Conception or design of the work; revising it critically for important intellectual content; final approval of the version to be published
AORS	Revising it critically for important intellectual content; final approval of the version to be published
MSPV	Revising it critically for important intellectual content; final approval of the version to be published
